# B-Myb Mediates Proliferation and Migration of Non-Small-Cell Lung Cancer via Suppressing IGFBP3

**DOI:** 10.3390/ijms19051479

**Published:** 2018-05-16

**Authors:** Xiaoyan Fan, Yitao Wang, Tinghui Jiang, Wei Cai, Yuelei Jin, Yulong Niu, Huifang Zhu, Youquan Bu

**Affiliations:** 1 Department of Biochemistry and Molecular Biology, College of Basic Medical Sciences, ChongQing Medical University, Chongqing 400016, China; fanxy@jumu.edu.cn (X.F.); wytao8899@126.com (Y.W.); jiangth@stu.cqmu.edu.cn (T.J.); caiwei5300@163.com (W.C.); jinyl@jmsu.edu.cn (Y.J.); zhu_huifang@126.com (H.Z.); 2 Molecular Medicine and Cancer Research Center, Chongqing Medical University, Chongqing 400016, China; yulong.niu@scu.edu.cn; 3 Department of Pathology, College of Basic Medical Sciences, Jiamusi University, Jiamusi 154007, China; 4 Department of Cell Biology, College of Basic Medical Sciences, Jiamusi University, Jiamusi 154007, China

**Keywords:** B-Myb, IGFBP3, NSCLC, proliferation, motility

## Abstract

B-Myb has been shown to play an important oncogenic role in several types of human cancers, including non-small-cell lung cancer (NSCLC). We previously found that B-Myb is aberrantly upregulated in NSCLC, and overexpression of B-Myb can significantly promote NSCLC cell growth and motility. In the present study, we have further investigated the therapeutic potential of B-Myb in NSCLC. Kaplan–Meier and Cox proportional hazards analysis indicated that high expression of B-Myb is significantly associated with poor prognosis in NSCLC patients. A loss-of-function study demonstrated that depletion of B-Myb resulted in significant inhibition of cell growth and delayed cell cycle progression in NSCLC cells. Notably, B-Myb depletion also decreased NSCLC cell migration and invasion ability as well as colony-forming ability. Moreover, an in vivo study demonstrated that B-Myb depletion caused significant inhibition of tumor growth in a NSCLC xenograft nude mouse model. A molecular mechanistic study by RNA-seq analysis revealed that B-Myb depletion led to deregulation of various downstream genes, including insulin-like growth factor binding protein 3 (IGFBP3). Overexpression of IGFBP3 suppressed the B-Myb-induced proliferation and migration, whereas knockdown of IGFBP3 significantly rescued the inhibited cell proliferation and motility caused by B-Myb siRNA (small interfering RNA). Expression and luciferase reporter assays revealed that B-Myb could directly suppress the expression of IGFBP3. Taken together, our results suggest that B-Myb functions as a tumor-promoting gene via suppressing IGFBP3 and could serve as a novel therapeutic target in NSCLC.

## 1. Introduction

Lung cancer is the most frequent cause of cancer-related deaths for patients in Western society; the five-year relative survival rate for lung cancer is around 18% [1]. Moreover, many studies have reported that lung cancer has become a major public health challenge as the incidence and mortality of lung cancer rapidly increases in developed countries, especially in China [2]. Non-small-cell lung cancer (NSCLC) comprises the majority of diagnosed lung cancer types, which is associated with a relatively poor 15% overall five-year survival rate. Despite there being many reports describing the therapy of NSCLC, the prognosis of the disease remains unfavorable. Thus, developing novel therapeutic strategies and innovative insights for NSCLC is necessary. 

B-Myb, also known as MYB proto-oncogene-like 2 (MYBl2), belongs to the Myb gene family that includes A-Myb and c-Myb [3,4,5]. B-Myb, acting as a transcription factor [6,7], is generally expressed in rapidly dividing cells [8], is crucially implicated in cell proliferation [9] and control of cellular differentiation [10], and has a vital role in guiding regulation of apoptosis [11] and tumor progression [12]. B-Myb has been shown to be overexpressed in many cancers, including colon cancer [13], hepatocellular carcinoma [14], breast cancer [15], T-cell lymphomas [16], and neuroblastoma [17]. Moreover, B-Myb is also associated with cancer patient prognosis with several types of cancers, including neuroblastoma [18], breast cancer [15], and acute myeloid leukemia [19]. We previously found that B-Myb is aberrantly upregulated in NSCLC, and overexpression of B-Myb can significantly promote NSCLC cell growth and motility [20]. However, little is known about the prognostic value and therapeutic potential of B-Myb in NSCLC.

Insulin-like growth factor binding protein 3 (IGFBP3) belongs to the IGFBP family, including six members, which mediate the bioactivity of insulin like growth factor (IGFs) and suppress mitogenic and antiapoptotic activity [21,22]. The mitogen-activated protein kinase (MAPK) and phosphatidyl inositol-3 kinase (PI3K) pathways, having important roles for cell proliferation and survival, were suppressed by overexpression of IGFBP3 [23]. Conversely, the low expression of IGFBP3 promotes cell survival and proliferation by activation of the PI3K/phosphorylated-protein kinase B (Akt) signaling pathway [24]. IGFBP3 is reported to be activated upon cellular senescence or mitotic quiescence [25,26]. Moreover, IGFBP3 is found to be linked with many factors, which include cytokines, hormones, peptide growth factors, vitamins, and chemotherapeutic agents [27,28]. 

In this study, we have found that B-Myb overexpression was related to shorter overall survival in lung cancer patients. A loss-of-function study demonstrated that knockdown of B-Myb significantly suppressed NSCLC cell growth, cell cycle progression, colony-forming ability, motility, and tumorigenesis in vivo. We also demonstrate that B-Myb regulated various downstream genes (e.g., *COL11A1*, *FLT4*, *SPARC*, *IDH2*, *PDK3*, and *IGFBP3*) and multiple pathways (e.g., the MAPK pathway) involved in cell proliferation, tumorigenesis, and metastasis. Moreover, we further demonstrated that IGFBP3 is an important downstream target gene of B-Myb, and that B-Myb activates extracellular signal-regulated kinases (ERK) and Akt signaling at least partially through inhibiting IGFBP3 in NSCLC cells. 

## 2. Results 

### 2.1. Prognostic Significance of B-Myb in NSCLC (Non-Small-Cell Lung Cancer) 

**Figure 1:**
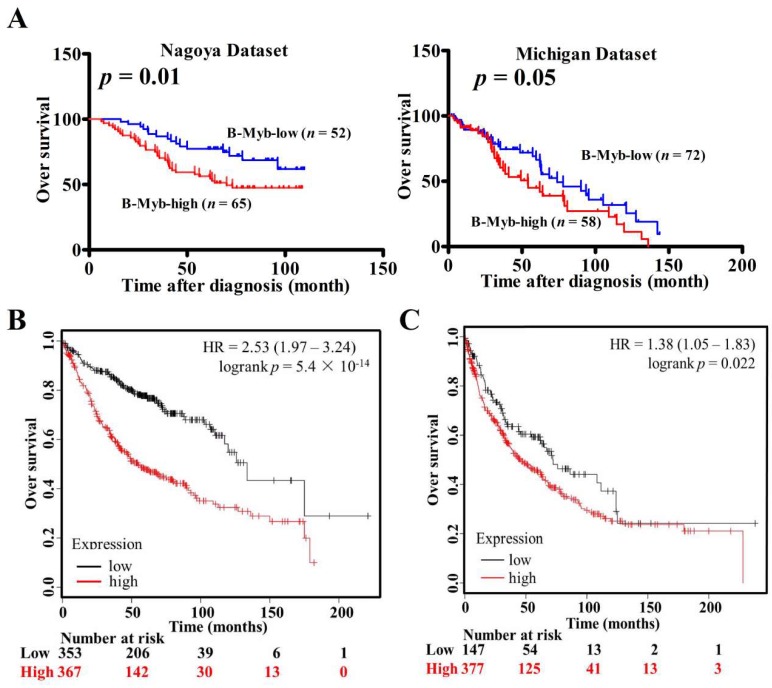
Prognostic significance of B-Myb in non-small-cell lung cancer (NSCLC). (**A**) Overall survival of lung cancer patients in the Nagoya lung adenocarcinoma (ADC) cohort and Michigan lung squamous cell carcinoma (SQCC) cohort. (**B**) Overall survival analysis of lung ADC patients by Kaplan–Meier plotter online tool. (**C**) Overall survival analysis of lung SQCC patients by Kaplan–Meier plotter online tool.

We previously found that B-Myb was upregulated in non-small-cell lung cancer (NSCLC). Moreover, the expression level of B-Myb was positively associated with tumor pathological grade, clinical stage, tumor, node, metastasis (TNM) classification, and lymph node metastasis [20]. In this study, we aimed at evaluating the prognostic value of B-Myb expression in silico using published microarray data, which are from primary tumors of lung cancer patients with follow-up information. As shown in Figure 1A, high expression of B-Myb was strongly associated with poor overall survival of lung cancer patients in the Nagoya University lung adenocarcinoma (ADC) cohort and Michigan University lung squamous cell carcinoma (SQCC) cohort (*p* < 0.05). In consistency with this observation, as shown in Figure 1B,C, online Kaplan–Meier plotter analysis [29] also revealed that B-Myb overexpression was negatively related to significant improvement in patient survival rates in lung ADC and SQCC. Moreover, univariate analyses revealed that high B-Myb expression was significantly associated with poorer survival in both cohorts (hazard ratio (HR) = 1.870, 95% confidence interval (CI) = 1.024–3.416, *p* = 0.042). Multivariate Cox regression analysis displayed that B-Myb expression was an independent prognostic factor for the Nagoya University cohort (HR = 1.789, 95% CI = 0.974–3.286, *p* = 0.043). In addition, lymph node metastasis was significantly related to poorer survival (*p* = 0.003) and the independent prognostic factor (*p* = 0.002) for the Nagoya University cohort (Table 1).

### 2.2. B-Myb Depletion Delays the Cell Cycle Progression and Inhibits Proliferation in Adenocarcinoma Cells (ADC)

**Figure 2:**
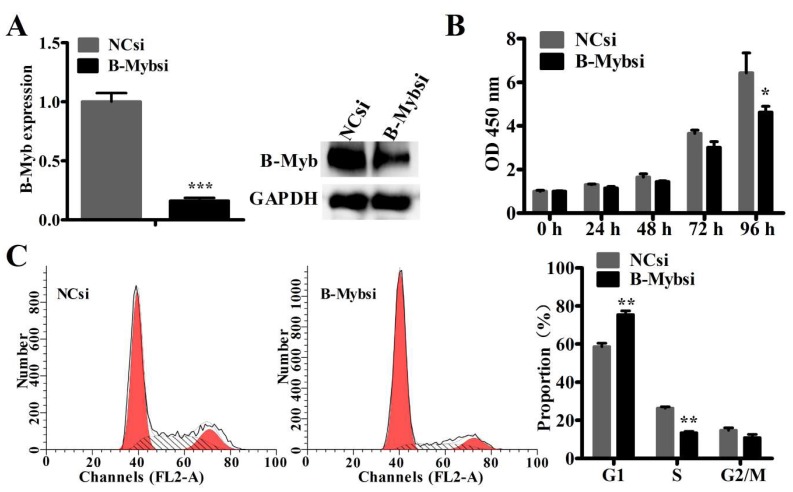
B-Myb depletion affects cell cycle progression and inhibits proliferation in A549 lung cancer cells. (**A**) A549 cells of small interfering RNA (siRNA)-mediated B-Myb silencing were transiently transfected with the negative control (NCsi) and B-Myb siRNA (B-Mybsi), respectively. Forty-eight and seventy-two hours after transfection, total RNA and whole cell lysates were respectively prepared and subjected to qRT-PCR and Western blot, and glyceraldehyde-phosphate dehydrogenase GAPDH as control proteins. (**B**) B-Myb depletion reduced cell proliferation. A549 cells were transiently transfected with negative control or B-Myb siRNA, and cell proliferation was then determined by cell counting kit-8 assay kits (CCK8) at the indicated time points. (**C**) B-Myb depletion delays G1–S phase transition. A549 cells were seeded on six-well plates and transfected with the indicated siRNAs, and twenty-four hours later, cells were collected and subjected to cell cycle analysis. All experiments were performed in triplicates. Data represent the mean ± standard deviation (SD). * *p* < 0.05, ** *p* < 0.01, *** *p* < 0.001.

To investigate the therapeutic potential of B-Myb in NSCLC, we depleted the B-Myb expression via small interfering RNA (siRNA)-mediated silencing in A549 lung cancer cell lines, and cell proliferation and cell cycle assays were subsequently performed. Quantitative RT-PCR and Western blot analysis showed that the B-Myb expression was significantly suppressed at both the mRNA and protein levels in A549 lung cancer cell lines (Figure 2A). B-Myb depletion resulted in a significant growth retardation compared with control siRNA from a later time point (96 h) in A549 cells (Figure 2B). Cell cycle analysis revealed that silencing B-Myb expression caused a remarkable G1 arrest in A549 cells (Figure 2C). Moreover, our previous study [20] showed that B-Myb depletion affects the cell cycle progression and inhibits proliferation in H1299 cells. These results suggested that B-Myb depletion mainly delays cell cycle progression and significantly inhibits proliferation in both A549 and H1299 cells.

### 2.3. B-Myb Depletion Reduces Motility in A549 Lung Cancer Cells

**Figure 3:**
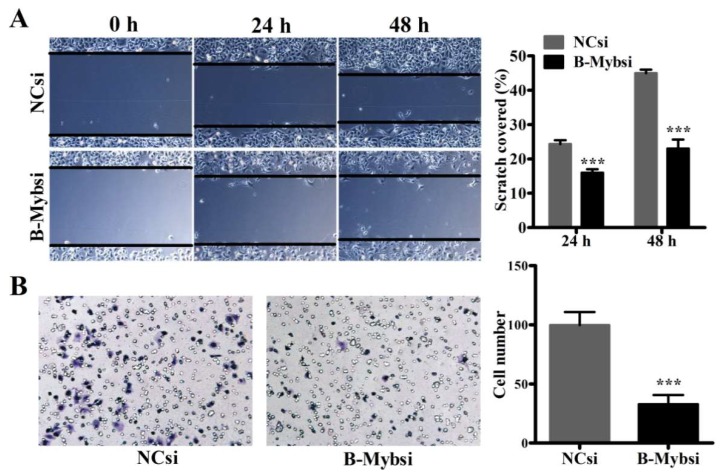
B-Myb knockdown inhibits motility in A549 lung cancer cells. A549 cells were transiently transfected with the negative control and B-Myb siRNA and then subjected to (**A**) would healing assay and (**B**) transwell migration assay as described in Materials and Methods section, respectively. Representative images (×200) (left) and quantification results (right) were shown for each assay. All experiments were performed in triplicates. Data represent the mean ± standard deviation (SD). *** *p* < 0.001.

Furthermore, we asked whether depletion of B-Myb expression regulates cell migration ability in A549 lung cancer cells. We performed wound healing and transwell migration assays to evaluate the cell migration ability. The wound healing assay suggested that siRNA-mediated silencing of B-Myb significantly decreased the rate of lateral migration of cells compared with the control cells (Figure 3A). In addition, the transwell migration assay showed that B-Myb knockdown also significantly suppressed the cell transmigration ability compared with the control cells (Figure 3B). Thus, these results strongly suggested that B-Myb might also regulate the cell motility of A549 lung cancer cells. 

### 2.4. B-Myb Depletion Inhibits Lung Tumorigenesis in Vitro and in Vivo

**Figure 4:**
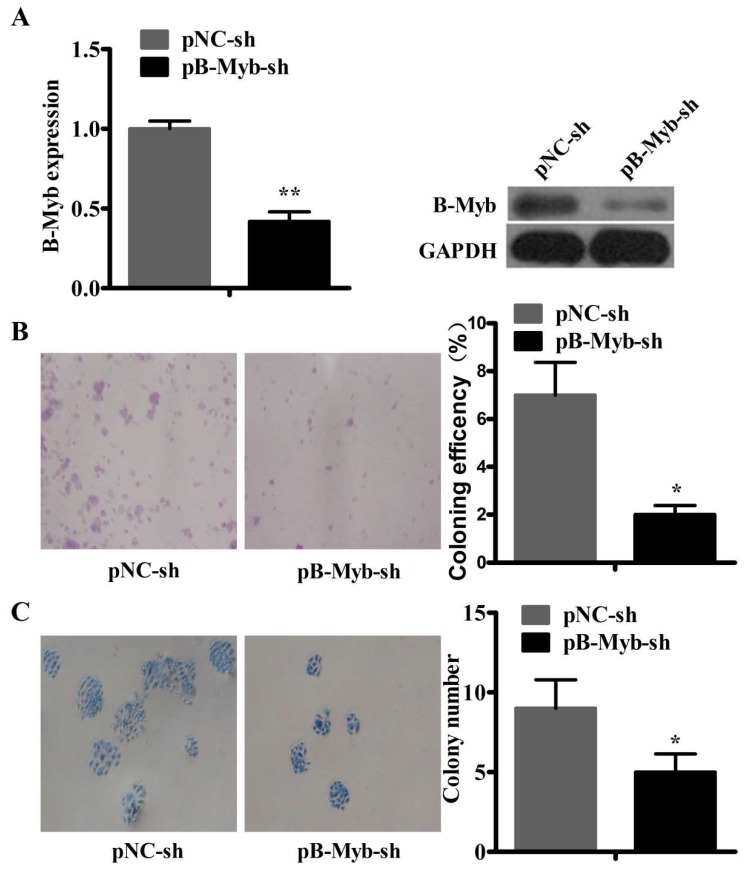
B-Myb knockdown inhibits the colony-forming ability of H1299 lung cancer cells. (**A**) Stable knockdown of B-Myb. H1299 lung cancer cells were transfected with pSuper-retro-sh empty vector and pSuper-B-Myb-sh vector. Cells were then selected with puromycin to establish the monoclonal stable negative control (pNC-sh) and B-Myb knockdown (pB-Myb-sh) cell lines. Expression of B-Myb was determined by qRT-PCR and Western blot analyses. (**B**,**C**) Stable knockdown of B-Myb inhibits colony formation. The stable negative control (pNC-sh) and B-Myb knockdown (pB-Myb-sh) H1299 cells were seeded on plastic plates for anchorage-dependent colony-formation assay (**B**) and soft agar for anchorage-independent colony-formation assay (**C**), respectively. Representative images (×200) (left) and quantification results (right) were shown for assay of B and C. All experiments were performed in triplicates. Data represent the mean ± SD. * *p* < 0.05, ** *p* < 0.01.

To further validate the function of B-Myb in the tumorigenesis of lung cancer, we established stable B-Myb knockdown cells in H1299 cells. Quantitative RT-PCR and Western blot results showed that the B-Myb expression was significantly decreased at both the mRNA and protein levels in the stable B-Myb knockdown cells (Figure 4A). Next, we analyzed the effect of B-Myb knockdown on the colony formation of H1299 lung cancer cells. As shown in Figure 4B,C, colony formation assays on plastic and soft agar revealed that B-Myb knockdown in H1299 cells significantly inhibited anchorage-dependent and -independent colony-forming ability compared with the control cells. 

**Figure 5:**
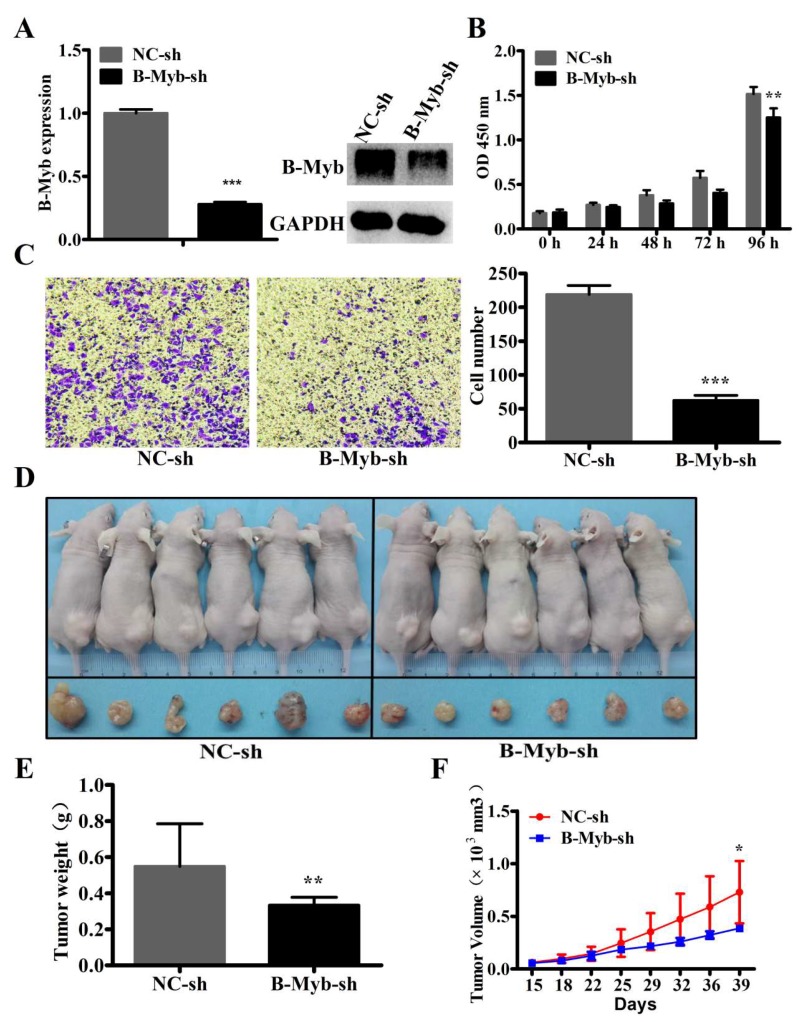
B-Myb knockdown inhibits the capability of tumorigenesis in vitro and in vivo. (**A**) A549 cells were treated with negative control and B-Myb knockdown lentiviral particles, and qRT-PCR and Western blot analyses were performed to examine the expression of B-Myb. (**B**) B-Myb knockdown decreases cell proliferation. Cell proliferation was determined by CCK8 in the stable negative control and B-Myb knockdown cells at the indicated time points. (**C**) B-Myb knockdown inhibits cell motility. Transwell migration assay was performed in the stable negative control and B-Myb knockdown cells. Representative images (×200) (left) and quantification results (right) were shown for this assay. (**D**,**E**) B-Myb knockdown inhibits lung tumor growth in vivo. Negative control cells and B-Myb knockdown cells were inoculated subcutaneously into the dorsal flanks of six nude mice, respectively. The tumor weight was measured at the end of the experiment. (**F**) The tumor growth curve. The tumor size was measured about twice a week, and a tumor growth curve was constructed. All experiments were performed in triplicates. Data represent the mean ± SD. * *p* < 0.05, ** *p* < 0.01, *** *p* < 0.001.

To examine whether silencing of B-Myb expression could inhibit tumorigenesis in vivo, we used A549 cells to establish stable cells with lentiviral particle B-Myb knockdown. Quantitative RT-PCR and Western blot analyses confirmed that the B-Myb expression was significantly decreased at both the mRNA and protein levels in the stable B-Myb knockdown cells (Figure 5A). In consistency with the results observed in the siRNA-mediated B-Myb depletion system, stable B-Myb knockdown also significantly decreased cell growth and motility in the lentivirus-mediated B-Myb depletion system, as evidenced by proliferation (Figure 5B) and transwell migration assays (Figure 5C). Subsequently, the stable B-Myb knockdown and the negative control cells were injected subcutaneously into the dorsal flank of nude mice, respectively. As shown in Figure 5D–F, stable knockdown of B-Myb expression remarkably suppressed the tumor growth in both tumor volume and tumor weight in the nude mice. 

### 2.5. RNA-Seq Analysis after siRNA-Mediated B-Myb Knockdown

**Figure 6:**
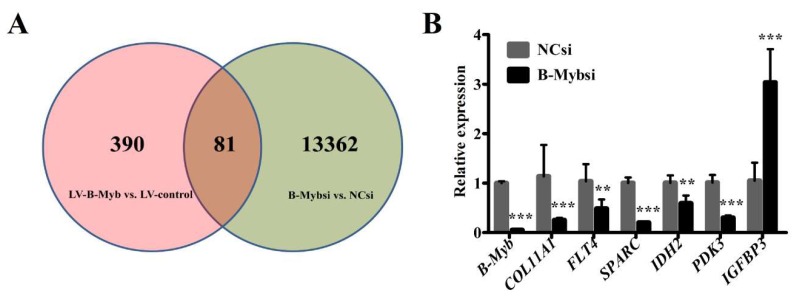
B-Myb knockdown affects various downstream genes and important pathways. (**A**) Venn diagram showing the distribution of DEGs that are unique or common between the two comparisons: LV-B-Myb vs LV-control H1299 cells [20] and B-Mybsi vs NCsi H1299 cells. (**B**) Validation of DEGs by qRT-PCR. qRT-PCR experiments were performed in triplicates. Data represent the mean ± SD. ** *p* < 0.01, *** *p* < 0.001.

To find genes and pathways affected by B-Myb depletion, we performed an RNA-sequencing (RNA-seq) assay to compare the differential gene expression profiles between B-Myb siRNA-transfected and negative control siRNA-transfected H1299 cells. According to RNA-seq analysis, the number of differentially expressed genes was 13,362. Among the differentially expressed genes (DEGs), 6343 genes were upregulated (ratio, >2.0), and 7019 genes were downregulated (ratio, <0.5) in response to B-Myb depletion. The DEGs were enriched in positive regulation of MAPK, ERK1, and ERK2 cascades; cell differentiation; cell proliferation; cell cycle; apoptosis; and so forth, (Table S1) as found via gene ontology analysis. Using the Kyoto Encyclopedia of Genes and Genomes databases, the significant signaling pathways of the DEGs affected by B-Myb depletion included the MAPK signaling pathway, cytokine–cytokine receptor interaction, transcriptional regulation in cancer, and so forth (Table 2). In a previous study [20], we had performed an RNA-seq assay between stable lentivirus (LV)-B-Myb overexpression and LV-control H1299 cells. We found that 81 genes were found to be dysregulated in both the past and present studies, shown as the overlapping segment in the Venn diagram (Figure 6A). Moreover, these genes, including *COL11A1*, *FLT4*, *SPARC*, *IDH2*, *PDK3*, *IGFBP3*, and so forth, were involved in proliferation, tumorigenesis, and metastasis. To further verify RNA-seq results, we performed a qRT-PCR assay to confirm the mRNA expression level of these genes in B-Myb siRNA-transfected H1299 cells compared with negative control H1299 cells. The qRT-PCR results corresponded well to the RNA-seq data (Figure 6B).

### 2.6. B-Myb Promotes Cell Proliferation and Migration by Targeting IGFBP3 in NSCLC Cells

**Figure 7:**
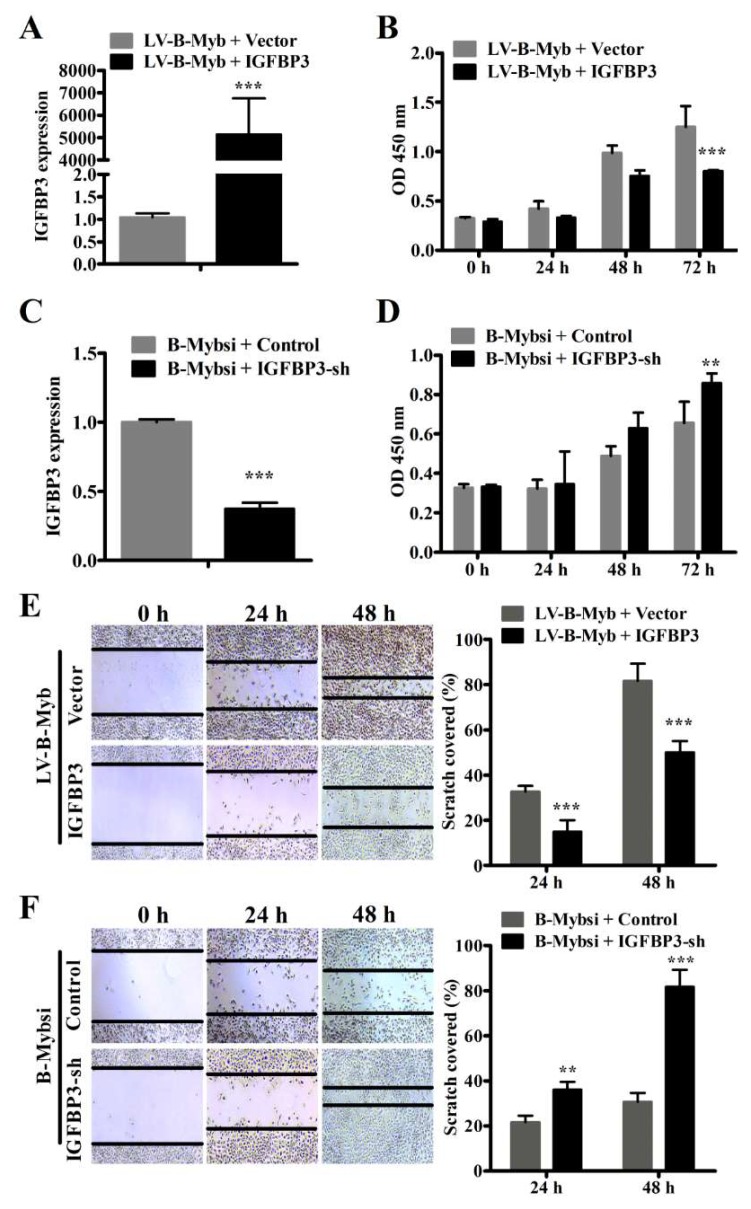
B-Myb regulated the proliferation and migration of H1299 lung cancer cells by targeting IGFBP3. (**A**) H1299 cells with stable B-Myb overexpression (LV-B-Myb) were transiently transfected with the empty vector and IGFBP3 plasmids, respectively. Forty-eight hours after transfection, total RNA was prepared and subjected to qRT-PCR. (**B**) IGFBP3 overexpression reduced cell proliferation induced by B-Myb overexpression. H1299 cells with stable B-Myb overexpression (LV-B-Myb) were transiently transfected with empty vector or IGFBP3 plasmids, and cell proliferation was then determined by cell counting kit-8 assay kits (CCK8) at the indicated time points. (**C**) H1299 cells with B-Myb depletion were transiently transfected with the empty vector and IGFBP3-sh plasmids, respectively. Forty-eight after transfection, total RNA was prepared and subjected to qRT-PCR. (**D**) IGFBP3 knockdown increased cell proliferation inhibited by B-Myb depletion. H1299 cells of B-Myb depletion were transiently transfected with empty vector or IGFBP3-sh plasmids, and cell proliferation was then determined by cell counting kit-8 assay kits (CCK8) at the indicated time points. (**E**) Wound healing assay was performed to detect whether IGFBP3 overexpression decreases the cell viability of H1299 cells induced by B-Myb overexpression. Representative images (×100) (left) and quantification results (right) were shown for this assay. (**F**) Wound healing assay was performed, revealing that IGFBP3 knockdown increases the cell motility of H1299 cells reduced by B-Myb depletion. Representative images (×100) (left) and quantification results (right) were shown for this assay. All experiments were performed in triplicates. Data represent the mean ± SD. ** *p* < 0.01, *** *p* < 0.001.

IGFBP3 has been shown to play an important role in proliferation, carcinogenesis, and ERK and Akt signaling pathways [23,24]. Thus, we decided to next investigate whether IGFBP3 acts as an important downstream target gene to mediate the oncogenic role of B-Myb in NSCLC cells. Firstly, we examined whether IGFBP3 overexpression could decrease the oncogenic effects of B-Myb on NSCLC cell proliferation. To this end, IGFBP3 expression plasmid and empty vector were transiently transfected to H1299 cells overexpressing B-Myb. Quantitative RT-PCR analysis confirmed the successful overexpression of IGFBP3 in cells (Figure 7A). IGFBP3 overexpression significantly suppressed the proliferation of H1299 cells induced by B-Myb (Figure 7B). Secondly, IGFBP3-siRNA and NC-siRNA were transiently transfected to H1299 cells that had been transfected with the B-Myb-siRNA. Quantitative RT-PCR analysis validated the successful knockdown of IGFBP3 in cells (Figure 7C). After transfection with IGFBP3-siRNA, proliferation of H1299 cells inhibited by B-Myb knockdown was markedly increased (Figure 7D). Moreover, in the wound healing assay, IGFBP3 overexpression inhibited the migratory capacity of H1299 cells induced by the B-Myb overexpression (Figure 7E). These results suggest that B-Myb promotes cell proliferation and migration by targeting IGFBP3 in H1299 cells. 

### 2.7. B-Myb Activates ERK and Akt Pathways via Targeting IGFBP3 in NSCLC Cells

**Figure 8:**
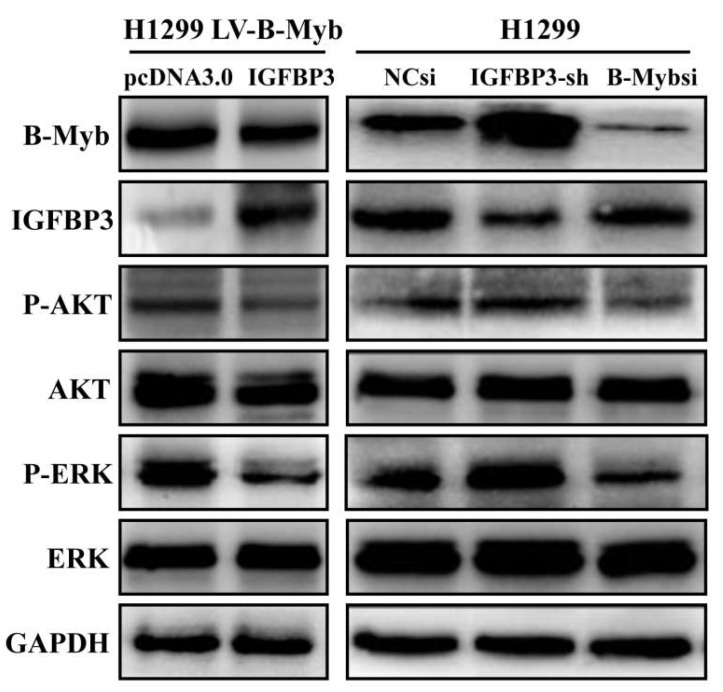
B-Myb activates ERK and Akt pathways via targeting IGFBP3. H1299 cells with stable B-Myb overexpression (H1299 LV-B-Myb) were transiently transfected with the empty vector and IGFBP3 plasmids, respectively. Forty-eight after transfection, whole cell lysates were prepared and subjected to Western blot analysis to detect the expression of the indicated protein. In addition, H1299 cells were transiently transfected with IGFBP3 shRNA plasmid or B-Myb siRNA. Forty-eight after transfection, whole cell lysates were prepared and subjected to Western blot analysis.

Our previous report showed that B-Myb overexpression could activate ERK and Akt pathways in NSCLC cells [20]. Therefore, we further investigated whether B-Myb regulates ERK and Akt pathways via IGFBP3. As shown in Figure 8, IGFBP3 overexpression significantly decreased the levels of phosphorylated ERK and Akt in the stable B-Myb expression cells. On the other hand, IGFBP3 knockdown increased the levels of phosphorylated ERK and Akt, whereas B-Myb knockdown decreased the phosphorylated ERK and Akt. Taken together, these results strongly suggest that B-Myb activates ERK and Akt signaling at least partially through inhibiting IGFBP3. 

### 2.8. B-Myb Inhibits IGFBP3 Promoter Activity

**Figure 9:**
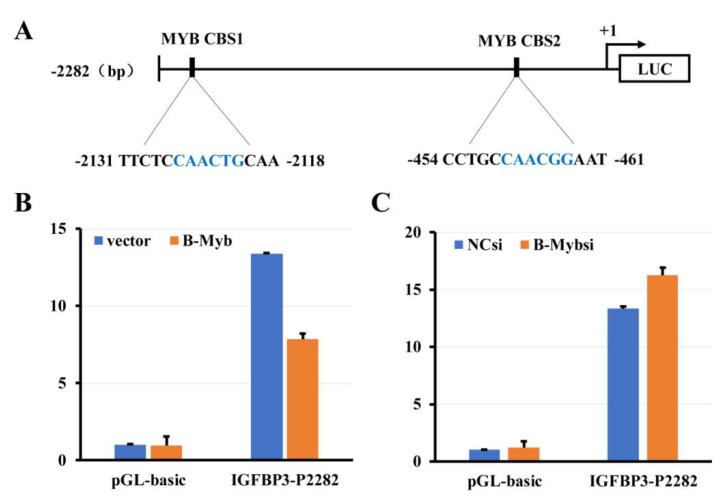
B-Myb suppresses IGFBP3 promoter activity. (**A**) Schematic diagram of the IGFBP3-P2282 luciferase (LUC) reporter construct. Two potential MYB-binding sites (MYB CBS1 and MYB CBS2) are shown. The transcription start site of the IGFBP3 gene is indicated as +1. (**B**) B-Myb overexpression inhibits IGFBP3 promoter activity. H1299 cells were transiently cotransfected in triplicate in 12-well plates with the B-Myb expression vector or empty control vector, and the IGFBP3-P2282 luciferase reporter or pGL3-basic control vector together with the Renilla luciferase reporter plasmid (pRL-TK) by using the Lipofectamine 2000 transfection reagent. Forty-eight hours after transfection, firefly and Renilla luciferase activities were measured by Dual Luciferase Assay System (Promega, Madison, WI, USA). Data obtained from a representative of at least three independent experiments were shown as the fold induction compared to the activity of cells transfected with the empty pGL3-basic vector. (**C**) B-Myb depletion enhances IGFBP3 promoter activity. The negative control (NCsi) or B-Myb siRNA (B-Mybsi) and the IGFBP3-P2282 luciferase reporter were transiently introduced into H1299 cells. Forty-eight hours after transfection, the luciferase activities were determined as in (**B**).

Finally, we asked whether B-Myb could directly regulate IGFBP3 in NSCLC cells. To this end, we firstly analyzed the putative transcription factor binding sites in the IGFBP3 promoter region, and found the existence of potential MYB-binding sites (t/cAACt/gG) in the IGFBP3 promoter (Figure 9A). To further verify whether B-Myb could directly suppress IGFBP3 gene transcription, H1299 cells were cotransfected with the B-Myb expression plasmid together with the IGFBP3-P2822 luciferase reporter plasmid. The luciferase reporter assay revealed that overexpression of B-Myb inhibits luciferase activities driven by the IGFBP3-P2822 promoter (Figure 9B). In a sharp contrast to B-Myb overexpression, B-Myb depletion enhances luciferase activities driven by the IGFBP3-P2822 promoter (Figure 9C). Collectively, our results strongly suggest that B-Myb directly suppresses IGFBP3 gene transcription. 

## 3. Discussion 

It has been well established that B-Myb expression is highly expressed in many types of cancers [13,15,30,31]. Additionally, B-Myb overexpression shows poor prognosis in patients with neuroblastoma [18], acute myeloid leukaemia [19], and breast cancer [15]. Tao et al. verified the B-Myb overexpression in breast cancer, particularly in advanced cancer, and demonstrated that B-Myb overexpression also predicts shorter overall survival of breast cancer patients [32]. Raschella et al. reported that B-Myb is overexpressed in neuroblastoma tumors, and that high expression level of B-Myb is an independent prognostic factor for poor neuroblastoma tumor patient survival [18]. Ren et al. confirmed that B-Myb expression is significantly increased in colorectal cancer (CRC) tissues compared to adjacent noncancerous tissues; that B-Myb overexpression is an independent prognostic factor for worse survival in colorectal cancer patients, as evidenced by Cox multivariate analysis; and that functional analysis showed that downregulation of B-Myb expression suppressed cell proliferation and motility, restricted cell cycle progression, and induced apoptosis [13]. Our previous studies verified that B-Myb is upregulated in non-small-cell lung cancer (NSCLC), and a gain-of-function study has verified that overexpression of B-Myb significantly promotes lung cancer cell growth, migration, and invasion [20]. In the present study, we firstly found prognostic significance of B-Myb in NSCLC, and a loss-of-function study demonstrated that downregulation of B-Myb significantly suppressed NSCLC cell growth, cell cycle progression, colony-forming ability, motility, and tumorigenesis in vivo. These results suggested that B-Myb is required for the accelerated proliferation and motility in NSCLC. Thus, the results of our study in NSCLC are consistent with the aforementioned reports obtained from other types of cancers. Moreover, the prognostic value of B-Myb expression was verified in NSCLC.

Previous studies have also indicated that the product of the B-Myb gene is a transcription factor that regulates the proliferation and progression of malignancies through suppression of the cell cycle and activation of genes and pathways corelated to tumorigenesis [3,4,8,33]. Aberrant B-Myb expression or amplification has been confirmed in different types of human cancers, verifying a role in tumorigenesis [31,34,35]. According to our recent observations [20], exogenous B-Myb overexpression in NSCLC promoted G1–S phase transition progression; caused transactivation of multiple downstream genes such as *CCNA1*, *COL11A1*, *COL6A1*, *MMP2*, *NID1*, and *FLT4*; and activated ERK and Akt signaling pathways. Consistently, our results in this study showed that knockdown of B-Myb caused a significant decrease in the number of S-phase cells. Moreover, RNA-seq analysis showed that knockdown of B-Myb leads to upregulation of different downstream genes such as *IGFBP3* and downregulation of various downstream genes such as *COL11A1*, *FLT4*, *SPARC*, *IDH2*, and *PDK3.* Previous reports showed that *SPARC* promotes cancer development in some cancers with highly metastatic characteristics, including melanoma and breast cancer [36,37], and was uncovered as the potential prognostic marker in several cancer types [38,39,40]. *IDH2* and *PDK3* have been shown to positively regulate the proliferation and tumorigenesis of various cancer cells [41,42,43]. It is worthwhile to mention that IGFBP3 overexpression suppresses the growth of NSCLC cells in vitro and in vivo, and leads to apoptosis in NSCLC cells by suppression of the signal transduction mechanism participating in cellular proliferation and survival [23]. It has been revealed that Akt and MAPK have significant inhibitory effects on apoptosis [44], and the function of Akt in survival has been confirmed in a study in which cells were exposed to different apoptotic stimuli [45,46,47]. Lee et al. reported that IGFBP3 overexpression inhibits the MAPK and Akt pathways, which have crucial roles in cell proliferation and survival [23]. Moreover, we also found that the differentially expressed genes (DEGs) affected by B-Myb depletion could be enriched in MAPK signaling pathways. Of note, our previous studies confirmed that B-Myb overexpression could enhance the activation of ERK and Akt signaling pathways in NSCLC [20]. Furthermore, our studies demonstrated that the expression level of IGFBP3 mRNA was negatively regulated by B-Myb expression, and that IGFBP3 negatively regulated the proliferation and migration of NSCLC induced by B-Myb differential expression. Furthermore, B-Myb depletion could suppress the activation of ERK and Akt signaling pathways though upregulation of *IGFBP3* in NSCLC, and B-Myb overexpression could promote the activation of ERK and Akt signaling pathways through inhibition of *IGFBP3*. In conclusion, our present study demonstrated for the first time that B-Myb is an independent prognostic marker and serves as a potential target in the diagnosis and/or treatment of NSCLC, and that B-Myb functions as a tumor-promoting gene by targeting IGFBP3 in NSCLC cells.

## 4. Materials and Methods 

### 4.1. Cell Culture 

The human lung cancer cell lines, H1299 and A549 (Chinese Academy of Sciences Shanghai cell bank, Shanghai, China), were maintained in RPMI 1640 (Hyclone, Logan, UT, USA) medium and DMEM (Hyclone) supplemented with 100 IU/mL penicillin, 100 mg/mL streptomycin, and 10% fetal bovine serum (FBS) (Invitrogen, Carlsbad, CA, USA), respectively. All the cells were cultured in a humidified incubator containing 5% CO_2_ at 37 °C. 

### 4.2. siRNA Synthesis and Transfection 

Small interfering RNAs against B-Myb (B-Mybsi) as well as the negative control (NCsi) were chemically synthesized by GenePharma (Shanghai, China). The sequences of the B-Myb siRNA are as follows: 5′-CAGACAAUGCUGUGAAGAATT-3′ (sense) and 5′-UUCUUCACAGCAUUGUCUGTT-3′ (antisense). The sequences of NC siRNA are as follows: 5′-UUCUCCGAACGUGUCACGUTT-3′ (sense) and 5′-ACGUGACACGUUCGGAGAATT-3′ (antisense). Knockdown of B-Myb expression was conducted using Lipofectamine RNAiMAX reagent (Invitrogen) according to the manufacturer’s instructions. Cells were collected and subjected to subsequent analysis 48 to 72 h after transfection. 

### 4.3. RNA Isolation and qRT-PCR

Total RNA was extracted using TRIzol reagent (Invitrogen) according to the manufacturer’s instructions. Reverse transcription of total RNA (500 ng) was performed for obtaining cDNA using PrimeScript^®^RT reagent Kit (TaKaRa, Otsu, Japan) according to the manufacturer’s instructions. qRT-PCR was performed using SYBR Premix Ex Taq™ (Perfect Real Time, TaKaRa). The primers sequences were provided in Table S2. Each experiment was repeated three times. 

### 4.4. Western Blot Analysis 

Cells were lysed in RIPA buffer including 1% protease inhibitor PMSF (Beyotime, Jiangsu, China). The proteins concentration was quantified by the bicinchoninic acid (BCA) protein assay kit (Thermo Scientific, Waltham, MA, USA). 40 μg of each protein was subjected to 8% SDS-PAGE, following transferral to the polyvinylidene fluoride (PVDF) membrane (Millipore, Burlington, MA, USA). The information of the antibodies used for Western blotting was provided in Table S3. The blots were visualized by the enhanced chemiluminescence (ECL; Bio-Rad Laboratories, Hercules, CA, USA). 

### 4.5. Cell Proliferation Assay

For the cell proliferation assay, 10 μL of cell counting kit-8 (CCK8; Dojindo, Tokyo, Japan) was added to the cells seeded in 96-well plates at a density of 0.2 × 10^4^ cells per well. The absorbance value (OD) of each well was measured at 450 nm at 0, 24, 48, 72, and 96 h after applying the CCK8 reagent according to the manufacturer’s instructions. The experiments were repeated three times.

### 4.6. Cell Cycle Assay

For the cell cycle assay, cells were collected by trypsin digestion and cell cycle assays were performed using the cell cycle kit (Key GEN, Nanjing, China) according to the manufacturer’s instructions. Cell cycle distribution was analyzed on a FACScan flow cytometer (BD Biosciences, San Jose, CA, USA).

### 4.7. Colony-Formation Assay

The anchorage-independent soft agar assay was conducted as described previously with minor modifications [33]. Anchorage-dependent colony-formation assay was performed in 6-well plates with a cell density of 2000 cells per well, and continuously cultured for ten days. Colonies were then fixed with 4% paraformaldehyde for 15 min and visualized by staining for 10 min with 0.5% crystal violet. 

### 4.8. Wound Healing Assay

The cells were seeded in 6-well plates and cultured. When the cells reached 80% confluence, artificial wounds were firmly scraped with a 10-μL pipette tip. Subsequently floating cells and debris were removed by washing the cells with PBS. The cells were then incubated in medium supplemented with 3% fetal bovine serum (Invitrogen), penicillin (100 IU/mL), and streptomycin (100 mg/mL). Initial gap widths (0 h) and residual gap widths at 24 h and 48 h after wounding were captured in the same wounded region under an inverted microscope (Olympus, Hamburg, Germany).

### 4.9. Transwell Cell Migration Assay 

For the transwell cell migration assay, A549 cells were seeded in the upper chamber of a transwell device (8-µm, Merck Millipore, Burlington, MA, USA) at a density of 2 × 10^4^ cells per well in serum-free medium, and 700 μL of medium containing 10% FBS was added to the bottom chamber. Thirty-six hours later, cells migrating into the lower surface of the chambers were fixed with methyl alcohol for 20 min and stained with 0.5% crystal violet for 10 min. Then, the cells on the upper surface of the filter were removed using a cotton swab. Five fields were imaged per transwell insert and were reviewed and photographed in five random fields, and the number of cells was counted using the particle-counting module of Photoshop CS5 (Adobe, San Jose, CA, USA).

### 4.10. Transfection 

Empty vector plasmids (pSuper-retro-sh) had been preserved in our laboratories, and short hairpin RNA (shRNA) was synthesized by Sangon Biotech (Shanghai, China) according to the sequence of control-siRNA or B-Myb-siRNA. The sequences of the siRNA used were listed in Materials and Methods, Section 4.2. Annealing shRNA was inserted into pSuper-retro-sh via enzyme digestion. Through transformation, selection, and plasmid extraction, the plasmids of negative control and silencing B-Myb gene were constructed well, and were named pControl-sh and pB-Myb-sh. H1299 cells seeded into 6-well plates were transiently transfected with 2 μg of these plasmids using Lipofectamine 2000 reagent (Invitrogen) according to the manufacturer’s instructions. After 6 h, the cells were seeded into 96-well plates at a density of about one cell per well and selected in the presence of 1.3 µg/mL puromycin for generating the control and stably silencing B-Myb clones. The survived puromycin-resistant cell clones were then continuously expanded and confirmed to construct the corresponding stable cell lines.

The IGFBP3 overexpression plasmid was a gift of Dr. Hiroshi Nakagawa (PA, USA) [48], and its control vector plasmid was preserved in our laboratories. Short hair IGFBP3 shRNA synthesized by Sangon Biotech (Shanghai, China) was inserted into the psi-LVRH1GP via enzyme digestion, and the IGFBP3 shRNA plasmid was constructed through transformation, selection, and plasmid extraction. The target sequence for IGFBP3 shRNA is as follows: 5′-GCACAGATACCCAGAACTT-3′. H1299 cells of B-Myb overexpression or B-Myb knockdown were transfected with corresponding plasmids to overexpress or knock down IGFBP3 using Lipofectamine 2000 reagent according to the manufacturer’s instructions, and incubated at 37 °C for 48 h.

### 4.11. Luciferase Reporter Constructs and Reporter Assays

The human IGFBP3 and (−2282 nucleotide (nt) to +56 nt)-luciferase promoters were gifted by Dr. T. Hanafusa (Okayama University, Okayama, Japan) [49]. Luciferase reporter constructs containing the potential MYB-binding sites were also generated using DNAMAN. All of the reporter constructs were validated by direct sequencing. For luciferase reporter analysis, cells were seeded in triplicate into 12-well plates and cotransfected with the indicated reporter vectors, pRL-TK vector (Promega) encoding Renilla luciferase together with the other vector, using Lipofectamine 2000 reagent (Invitrogen). Forty-eight hours later, cells were lysed, and their luciferase activities were measured using the dual luciferase assay system (Promega) as described previously.

### 4.12. Lentiviral Infection and Construction of Stably Silencing B-Myb Gene A549 Cell Lines 

Lentiviral vectors (LV-B-Myb-RNAi and LV-control-RNAi) were obtained from GeneChem (Shanghai, China) and respectively infected into A549 cells according to the manufacturer’s instructions. Infected derivative cells stably expressing shRNA were selected in the presence of 0.5 µg/mL puromycin for 10 days, then named B-Myb-sh and NC-sh.

### 4.13. Tumor Xenografts

All procedures conformed to the legal mandates and guidelines of the Laboratory Animal Center of Chongqing Medical University for the care and maintenance of laboratory animals. 3 × 10^7^ stable control or B-Myb silencing cells were subcutaneously injected into the dorsal flank of four- to six-week old nude mice (BALB/c). Six mice were used for each group. The xenograft size was measured every four days by a Vernier caliper along two perpendicular axes. The volume of the xenograft was calculated following the formula: volume = 1/2 × length × width^2^. Eight weeks after injection, the mice were killed and the tumor specimens were weighed. All experimental procedures involving animals were conducted based on laboratory animal protocols approved at 3 May 2016 by the Laboratory Animal Center of Chongqing Medical University (No. 20160503).

### 4.14. RNA-Seq Analysis 

RNA-seq analysis was performed as described previously [20].

### 4.15. Prognostic Analysis of B-Myb in Lung Cancer Patients

Prognostic analysis of B-Myb in lung cancer patients were performed as described previously [49]. The Nagoya lung adenocarcinoma (ADC) and Michigan lung squamous cell carcinoma (SQCC) datasets were downloaded from the public gene expression omnibus (GEO) database (GSE4573 and GSE13213 [29]. In addition, the prognostic value of B-Myb expression in lung ADC and SQCC was also evaluated by an online tool: Kaplan–Meier plotter analysis [29]]. 

### 4.16. Lentivirus-Mediated Establishment of Stable B-Myb Overexpression Cell Line 

According to the method described previously [20], the lentivirus-mediated establishment of a stable B-Myb overexpression cell line was accomplished.

### 4.17. Statistical Analysis 

All of the statistical analyses were performed using the SPSS16.0 software (SPSS INC, Chicago, IL, USA). Statistical significance among different groups in the experiments was tested by ANOVA and unpaired *t*-test. Overall survival curves were estimated by the Kaplan–Meier method and compared among groups by log-rank test. The Cox proportional hazards regression model was applied to perform univariate and multivariate regression analyses, which calculate the hazard ratios (HRs) and their 95% confidence intervals (95% CIs) for each variable and analyze independent factors affecting prognosis. All comparisons were carried out in two-tailed *t*-tests, and *p* values of <0.05 were taken to be statistically significant.

## Figures and Tables

**Table 1:** Univariate and multivariate analysis of different prognostic parameters for lung adenocarcinoma patients in the testing cohort and validation cohort.

Variable	Multivariate Analysis		Univariate Analysis	
	HR (95% CI) ^a^	*p* Value ^b^	HR (95% CI) ^a^	*p* Value ^b^
117 lung cancer patients				
B-Myb (High/Low)	1.789 (0.974–3.286)	0.043	1.870 (1.024–3.416)	0.042
AGE (≥60/<60)	1.145 (0.627–2.090)	0.660		
SEX (Male/Female)	1.456 (0.808–2.623)	0.211		
T (T1, T2/T3, T4)	1.785 (0.82–3.886)	0.144		
N (N0/N1, N2)	2.533 (1.403–4.573)	0.002	2.413 (1.347–4.326)	0.003

^a^ HRs and 95% confidence intervals (CIs) were calculated using univariate or multivariate Cox proportional hazards regression in SPSS16.0. ^b^ *p* values were calculated using univariate or multivariate Cox proportional hazards regression in SPSS16.0. *p* values <0.05 were considered to indicate statistical significance.

**Table 2:** Important pathway and genes affected by B-Myb knockdown.

Pathway	Counts	Dysregulated Genes	*p* Value
**MAPK signaling pathway**	59	*ACVR1C*, *CASP1*, *CASP14*, *CASP5*, *CD14*, *COL11A1*, *DDIT3*, *DUSP10*, *EGFR*, *ELK4*, *FAS*, *FASLG*, *FGF1*, *FGF10*, *FGF11*, *FGF13*, *FGF17*, *FGF19*, *FGF22*, *FGF8*, *FGFR4*, *FLT4*, *FOS*, *GADD45A*, *GNA12*, *HSPA9B*, *IL1A*, *IL1B*, *IL1R1*, *JUN*, *MAP2K5*, *MAP2K6*, *MAP3K14*, *MAP3K8*, *MAPK11*, *MAPK6*, *MAPK8IP2*, *MRAS*, *NFATC2*, *NTF3*, *NTRK1*, *PDGFRA*, *PLA2G10*, *PLA2G4A*, *PRKCB1*, *PRKX*, *PRKY*, *PTPN7*, *PTPRR*, *RAP1A*, *RASGRF1*, *RASGRP3*, *RASGRP4*, *RPS6KA4*, *STMN1*, *TGFB2*, *TMEM37*, *TNF*, *TP53*	3.2 × 10^−3^
**Cytokine-cytokine receptor interaction**	83	*TNFSF15*, *TNFSF4*, *TNFRSF4*, *MPL*, *TNF*, *HGF*, *CCL27*, *TNFSF12*, *CCL2*, *TNFRSF8*, *TNFSF10*, *TNFSF8*, *TNFSF9*, *IFNLR1*, *TNFSF14*, *TNFRSF6B*, *CCL5*, *KITLG*, *TNFRSF19*, *IFNA1*, *CXCR4*, *TNFRSF17*, *CXCR2*, *IL7R*, *IL12RB*, *IL12B*, *IL13*, *IL23A*, *IL11RA*, *IL12A*, *CTF1*, *FLT1*, *FLT3*, *IL1A*, *FLT4*, *TNFRSF1B*, *CD27*, *TNFRSF12A*, *CXCR6*, *CCL20*, *EDAR*, *CCL21*, *IFNA13*, *IL10*, *TNFRSF13C*, *CSF1R*, *LTA*, *CSF1*, *IL3RA*, *CNTF*, *EGFR*, *IL2RG*, *IL2RB*, *TNFRSF9*, *OSM*, *IL15RA*, *IFNE*, *CSF2RA*, *TGFB2*, *CXCL13*, *LTB*, *CXCL11*, *CCR9*, *NGFR*, *IL1B*, *AMHR2*, *IL1R1*, *AMH*, *IL20RB*, *TNFRSF25*, *IL22RA1*, *LTBR*, *CXCR3*, *IL20RA*, *PDGFRA*, *GHR*, *CCR10*, *FAS*, *LIF*, *FASLG*, *IFNW1*, *IL4*, *TSLP*	6.39 × 10^−6^
**Transcriptional regulation in cancer**	57	*PAX8*, *HIST1H3F*, *ELK4*, *ZBTB16*, *ITGAM*, *CEBPB*, *TCF3*, *FCGR1A*, *SUPT3H*, *IGFBP3*, *PLAT*, *SIX1*, *MMP*, *3 HIST2H3C*, *FLT1*, *GRIA3*, *SPI1*, *ID2*, *FLT3*, *MDM2*, *TFE3*, *UTY*, *SPINT1*, *CD14*, *CDK14*, *HIST1H3G*, *HIST1H3E*, *HIST1H3C*, *HIST3H3*, *HIST2H3A*, *HIST1H3J*, *CSF1R*, *TSPAN7*, *PBX1*, *IL2RB*, *RUNX1T1*, *ERG*, *TAF15*, *CDKN2C*, *ELANE*, *DDIT3*, *ETV7*, *NGFR*, *REL*, *BCL2A1*, *TMPRSS2*, *TP53*, *ATF1*, *HIST2H3D*, *NUPR1*, *EYA1*, *HDAC*, *1 SLC45A3*, *CCNT2*, *NTRK1*, *LMO2*, *SSX1*	1.24 × 10^−3^
**cAMP signaling pathway**	57	*JUN*, *ADCY8*, *CALM2*, *ADCY7*, *NPY*, *PTGER3*, *CALM1*, *HCAR3*, *GRIA4*, *ATP1A4*, *ATP1B1*, *PPP1R12A*, *GRIN1*, *GRIN2A*, *GRIN2B*, *CACNA1D*, *CFTR*, *VAV1*, *TNNI3*, *GRIA3*, *CACNA1F*, *GRIA1*, *ADCY4*, *ATP1A3*, *ATP1A2*, *HTR1B*, *CREB5*, *TIAM1*, *HCAR2*, *PIK3R5*, *ADORA2A*, *PDE3B*, *PDE4A*, *SSTR1*, *PDE4D*, *HTR6*, *CHRM2*, *ADCY2*, *ADCY1*, *PDE3A*, *AMH*, *NPR*, *1 RAP1A*, *GLI1*, *PIK3R3*, *CAMK2A*, *CAMK2D*, *HCAR1*, *GLI3*, *PTCH1*, *VIPR2*, *HCN4*, *ACOX1*, *CNGA1*, *CALM3*, *FOS*, *GRIN3A*	0.045166
**Rap1 signaling pathway**	60	*ADCY8*, *ANGPT4*, *CALM2*, *ADCY7*, *PRKCB*, *SIPA1L1*, *CALM*, *1 HGF*, *FGF8*, *ANGPT2*, *ITGAM*, *PDGFD*, *KITLG*, *FGF1*, *F2RL3*, *RAPGEF2*, *GRIN1*, *GRIN2A*, *GRIN2B*, *MAPK11*, *ID1*, *FLT1*, *FLT4*, *MAP2K6*, *FGFR4*, *ADCY4*, *LPAR5*, *EFNA2*, *P2RY1*, *FGF17*, *FGF10*, *RASGRP3*, *ITGB2*, *FGF11*, *SKAP1*, *TIAM1*, *FGF13*, *FGF19*, *PIK3R5*, *ADORA2A*, *CSF1R*, *CSF1*, *FPR1*, *EGFR*, *LAT*, *RASSF5*, *ADCY2*, *ADCY1*, *MRAS*, *NGFR*, *CNR1*, *RAP1A*, *PIK3R3*, *FGF22*, *RALB*, *PDGFRA*, *TEK*, *CALM3*, *FYB*, *CDH1*	0.045802

*p* values < 0.05 were considered to indicate statistical significance.

## Supplementary Materials

Supplementary materials can be found at http://www.mdpi.com/1422-0067/19/5/1479/s1.

## Abbreviations

NSCLC	non-small-cell lung cancer
Mybl2	MYB proto-oncogene-like 2
COL11A1	collagen type XI alpha 1 chain
FLT4	fms-related tyrosine kinase 4
SPARC	secreted protein acidic and cysteine rich
IDH2	isocitrate dehydrogenase (NADP(+)) 2
IGFBP3	insulin-like growth factor binding protein 3
PDK3	pyruvate dehydrogenase kinase 3
