# Supplementary material for: B-Myb Mediates Proliferation and Migration of Non-Small-Cell Lung Cancer via Suppressing IGFBP3
Source: Int J Mol Sci. 2018 May 16;19(5):1479. doi: 10.3390/ijms19051479 (PMC5983693; doi:10.3390/ijms19051479)
Supplement: Supplementary file 1 [file ijms-19-01479-s001.zip › Supplementary Files/Table S1.docx]

**Table S1.** Gene Ontology Enrichment Analysis on genes affected by B-Myb knockdown

| Gene Ontology term | Counts | Genes annotated to the term | *p* value |
| --- | --- | --- | --- |
| transcription factor activity, sequence-specific DNA binding | 277 | *ZFP69B, HIF1A, ZSCAN2, MYBL2, UBP1, TAF5, PHOX2A, MEOX2, ZSCAN10, ZNF540, DMRTC1B, ELF2, ZNF888, NFIX, ID3, SMAD5, NFIL3, NFIC, ZNF174, NFIB, NR1I2, NR2C2, SMAD6, FOXS1, FOXC2, BTBD8, ZNF281, ZNF546, FOXJ1, ASCL1, MEF2BL3, MBTL4, NFATC2, ZBED1, TSC22D1, ZNF155, GTF2H3, PAX8, ZBTB25, ZNF415, MZF1, ZNF43, ZNF45, SALL3, SCML2, BATF3, TEAD2, NEUROD1, ZNF880, HOXD8, PLAGL1, TBX4, ZNF91, ID1, HOMEZ, IRF9, ZNF793, E2F8, YEATS4* etc | 3.1 × 10^-5^ |
| cell differentiation | 134 | *ZNF431, SPATA31A7, ELK3, ELK4, ZNF268, MEIG1, TNFSF12, KLRC4-KLRK1, COL19A1, CCDC169-SOHLH2, SH2D2A, HIST1H1T, MYCBPAP, NKX2-5, RGS20, ZIC1, TSSK3, ZGLP1, USP42, CATSPER2, SLC9C1, SPIB, FLT1, FLT3, FOXN2, ZSCAN2, MYBL1, MYBL2, EDAR,DLL1, NKAPL, HLX, ACSBG2, RBM11, SPATA9, ASF1B, CSF1, ATOH8, SLFN5, ROBO4, NR1D1, EYA4, APAF1, GNA12, GNA13, ANXA13* etc | 3.7 × 10^-3^ |
| regulation of apoptotic process | 68 | *PAX8, FRS2, CIDEA, DYNAP, TNFRSF4, BIRC3, IFT57, GDF7,NDRG1, ANP32D, INHBC, CRADD, CARD6, CARD1, IGFBP3, SKIL, BCL3, GAS1, MAGED1, BID, CASP5, FLT3, RASSF3, CASP1, TNFRSF1B, C8orf44-SGK3, NME4, TP53INP1, PERP, SGK1, ETS1, MSTN, ALK, CARD9, ANP32E, EGR1, ROBO4, PRDX2, APAF1, SGK3, TNFRSF9, NOS1AP, RASSF5, BOK, BBC3, NTF3, NGFR, BEX2, LEFTY1, TNFRSF25, SARM1, DLG5* etc | 4.1 × 10^-3^ |
| regulation of cell proliferation | 56 | *JUN, TFAP2C, PLCD1, SRMS, TNFRSF4, TNF, NDRG1, TNFRSF6B, SIX3, RAPGEF2, MZB1, ERBB3, BID, PLA2G4A, TFRC, PTGS1, TNFRSF1B, FA2H, C8orf44-SGK3, CD27, SAT1, PPP1R9B, GUCY2C, SGK1, EGLN3, GKN2, TEC, SHH, SGK3, ITK, CFDP1, CXCL13, TRNP1, BRICD5, CXCL11, KCNH1, NGFR, LTBR, LGR5, TNFRSF25, CSK etc* | 0.027126255 |
| positive regulation of ERK1 and ERK2 cascade | 50 | *JUN, PTPN11, HMGB1, TNF, FGF8, CCL2, PDGFD, CCL5, FGF1, PTPN22, HAVCR2, RAPGEF2, ALOX15, ERBB4, GPR55, FGFR4, FLT4, NPNT, P2RY1, CCL20, NQO2, FGF10, CCL21, NRP1, NELFE, CCL22, FGF19, CSF1R, BMPER, EGFR, CCL24, PYCARD, SEMA7A, SYT14P1, RAP1A, C5AR2, PTEN, SPRY2, PDGFRA, CAMK2D, TEK, BMP4, SERPINF2, GPR183, GLIPR2, PTK2B, GPNMB, HCRTR1, HAND2, NTRK1* | 0.04880807 |
| positive regulation of MAPK cascade | 26 | *SOX2, HMGB1, TNFRSF4, OSM, TNFRSF6B, C1QTNF1, ZNF622, IGFBP3, AR, LAMTOR1, BANK1, NGFR, ALOX12B, TNFRSF25, ADRA1B, KISS1, FLT1, FLT3, FLT4, IGFBP4, TNFRSF1B, TRIM5, CD27, FAS, FGF10, LIF* | 0.044873059 |
| crest cell migration | 22 | *ERBB4, ISL1, EDNRB, SHH, SEMA6B, SEMA6A, SEMA3D, SEMA3B,HIF1A, SEMA4D, PHACTR4, SEMA4G, SEMA4B, SEMA3C, NRTN, SEMA3G, RET, KITLG, SEMA5A, FGF19, SEMA4A, SEMA7A* | 5.11 × 10^-4^ |
| negative regulation of cell cycle arrest | 9 | *CCND1, MLXIPL, TFAP4, FGF10, ZNF268, FZD9, HMGN5, MDM2, RASSF1* | 0.043265852 |
