# Supplementary material for: B-Myb Mediates Proliferation and Migration of Non-Small-Cell Lung Cancer via Suppressing IGFBP3
Source: Int J Mol Sci. 2018 May 16;19(5):1479. doi: 10.3390/ijms19051479 (PMC5983693; doi:10.3390/ijms19051479)
Supplement: Supplementary file 1 [file ijms-19-01479-s001.zip › Supplementary Files/Table S2.docx]

**Table S2.** List of primer sequences used for qRT-PCR analysis

| Gene name | Primer sequences |
| --- | --- |
| *GAPDH* | GAPDH F (833): ACCTGACCTGCCGTCTAGAA  GAPDH R (1060): TCCACCACCCTGTTGCTGTA |
| *B-Myb* | B-Myb F (1067): AGAAACGAGCCTGCCTGCCTTACA  B-Myb R (1220): AGATGGTTCCTCAGGGAGGT |
| *COL11A1* | COL11A1 F (1275): GCCTGGTATGCTTGTCGAAG  COL11A1 R (1508): CCTGAGCAGAGATGGTTGG |
| *FLT4* | FLT4 F (2870): AAGTACGGCAACCTCTCCAA  FLT4 R (3086): CCTCAGCTTCTTGGTCTGGA |
| *SPARC* | SPARC F (781): TGCGGGTGAAGAAGATCCAT  SPARC R (1030): TTGTCCAGGTCACAGGTCTC |
| *IDH2* | IDH2 F (609): TGCTTCCAGTATGCCATCCA  IDH2 R (841): TCATAGTTCTTGCAGGCCCA |
| *PDK3* | PDK3 F (1146): CTACTGCTCCTAGACCCAGC  PDK3 R (1382): CAGGCGTGGTCTTGTAATGG |
| *IGFBP3* | IGFBP3 F (613): TCTGATCCCAAGTTCCACCC  IGFBP3 R (785): TCCATTTCTCTACGGCAGGG |
