# Supplementary material for: B-Myb Mediates Proliferation and Migration of Non-Small-Cell Lung Cancer via Suppressing IGFBP3
Source: Int J Mol Sci. 2018 May 16;19(5):1479. doi: 10.3390/ijms19051479 (PMC5983693; doi:10.3390/ijms19051479)
Supplement: Supplementary file 1 [file ijms-19-01479-s001.zip › Supplementary Files/Table S3.docx]

| **Protein**  **name** | **Manufacture**  **(cat. number)** | **Applications**  **(working dilution)** | **Website Link** |
| --- | --- | --- | --- |
| GAPDH | Xianzhi Bio  (AB-P-R 001) | IB (1:5000) | http://www.goodhere.com/showproduct.asp?id=320&classid=34&nid=2 |
| B-Myb | Santa Cruz  (N-19):sc-724 | IB (1:500) | <http://www.scbt.com/datasheet-724-b-myb-n-19-antibody.html> |
| antiRabbit secondary antibody | Abgent  (ASS1009) | IB (1:5000) | http://www.abgent.com/products/ASS1009-Goat-Anti-Rabbit-IgGHL-MouseHuman-ads-HRP-Secondary-Antibody |

**Table S3.** Antibodies used in the present study

IB: immunoblot; GAPDH: glyceraldehyde-3-phosphate dehydrogenase.
